# Peer Attachment and Proactive Socialization Behavior: The Moderating Role of Social Intelligence

**DOI:** 10.3390/bs12090312

**Published:** 2022-08-29

**Authors:** Ting Nie, Yanying Zheng, Yiying Huang

**Affiliations:** School of Business, Macau University of Science and Technology, Macau 999078, China

**Keywords:** peer attachment, peer trust, peer communication, peer alienation, core self-evaluation, proactive socialization behavior, social intelligence

## Abstract

Proactive socialization involves an active attempt to integrate into an organization, which can help an individual complete the transition from student to employee. This study—conducted via a survey involving college graduates (one year after graduation)—explores the peer attachment influence mechanism on proactive socialization behaviors and the moderating effects of social intelligence. The results of the empirical analysis show that core self-evaluation has a mediating effect between peer attachment and individual proactive socialization behavior. Peer trust and peer communication can improve individual proactive socialization behavior by enhancing core self-evaluation, but peer alienation may reduce core self-evaluation and inhibit individual proactive socialization behavior in the workplace. Social intelligence has a moderating mediating role between peer attachment, core self-evaluation, and proactive socialization behavior. High social intelligence may enhance the indirect influence of peer trust and communication on proactive socialization behavior through core self-evaluation and weaken the indirect influence of peer alienation on proactive socialization behavior through core self-evaluation. In recruitment and selection, organizations can predict the proactive socialization behaviors of candidates by investigating their peer relationships, and can also strive to create a harmonious working atmosphere and relationship to help new employees integrate into the organization.

## 1. Introduction

Organizational socialization is an important process in which individuals change roles from students to employees. It enables new employees to obtain the necessary knowledge and competencies, clarifies their roles to complete their expected tasks, helps them gain recognition from colleagues, and establishes cooperative relationships (a key source of social support for new employees) [1]. Many studies focus on verifying the dominant roles of organizations in the socialization process, and employees are only passive responders [2]. Organizations can influence new employee perceptions and behaviors through socialization strategies, which can improve a new employee’s job satisfaction and work performance, and reduce future turnover ‘behaviors’ [3]. New employees are not completely passive; some will take the initiative to understand and adapt to the new environment. They could avoid isolation through spontaneous information gathering, feedback seeking, and relationship building, which can help them accumulate interpersonal resources and acquire the support they need to achieve self-improvement [4]. Ashford and Black [5] defined these various active social behaviors adopted by new employees in order to adapt to new jobs and organizations as proactive socialization behaviors. Compared with organizational socialization, proactive socialization behaviors place more emphasis on the sense of personal control and the promotion of self-adaptation to the new environment [6]. Previous studies have confirmed that proactive socialization behavior is closely related to personality traits, organizational climate, and leading styles [7], but it rarely involves the personal social networks of new employees.

When individuals grow from adolescence to emerging adulthood, parental attachment is gradually replaced by peer attachment [8]. The influence of peers is growing, especially in the period of time after graduating from university, the interaction between classmates and friends is more frequent, which will have a significant impact on whether new employees can consciously integrate into the organization and display proactive socialization behaviors. Meanwhile, individuals with higher social intelligence are better at utilizing these interpersonal resources, as well as socializing and career development [9]. The influence mechanism of peer attachment on individual proactive socialization behaviors and the moderating effects of social intelligence will be further explored in the study.

## 2. Research Hypotheses

Attachment is an important bond that exists between individuals [10,11]. As adolescents grow up, the attachment relationship shifts from attachment with parents to attachment with peers. They will be more willing to exchange information with their peers [8]. Peer attachment is usually assessed in three dimensions: trust, communication, and alienation. Peer trust emphasizes mutual trust and respect for each other’s needs and desires; peer communication refers to the perceived quality of participation, responsiveness, and the state of verbal communication; peer alienation involves feelings of anger, isolation, and separation from peers [12].

Different peer attachments are predictive of personal perceptions and behaviors. Secure peer attachment is built on trust, resulting in mutual understanding and high-quality communication [13,14]. Insecure peer attachment is generally accompanied by alienation and isolation from peers [15]. Peer attachment relationships are critical for adolescent social behaviors and social emotions [16]. High-quality peer relationships can promote the development of individual trait strengths, thereby enhancing their self-regulation, sociability, and positive prosocial behaviors [16,17]. As adolescents grow into adulthood, emerging adults spend more time with their peers, and individuals with good peer relationships have higher social well-being [18]. Peer relationship is important in the behavioral development of newcomers entering an organization. Positive peer-support relationships are helpful for the social integration of emerging adults [9]. Newcomers promote the development of social integration through peer-to-peer competition or cooperation in friendship group networks [19]. When newcomers form higher quality or higher intensity bonds with their peers over a period of time, they may gain more work-related information. Peer perception helps newcomers to better understand and define their role boundaries with their peers; thus, it is a unique mechanism to promote social adjustment [20].

To gain a more complete understanding of an organization, new members may engage in proactive socialization behaviors, such as acquiring information on organizational policies and building social networks within companies [5]. Proactive socialization behaviors typically fall into four categories: seeking information (e.g., understanding the organization policy), obtaining feedback from supervisors (e.g., asking for advice or suggestions), negotiating for a job change (e.g., negotiating with others about ideal work), and general social activities (e.g., attending company social gatherings) [21]. Numerous studies have shown that individual factors (employee proactive personality, extroversion, openness, etc.), organizational factors (organizational socialization strategies, training or social gatherings, etc.), and peer factors (relationships with colleagues, leadership styles, and mentors, etc.) all have significant effects on individual proactive socialization behaviors [7,21,22], which can help newcomers understand and adapt to the working environment [23], and lead to positive work outcomes, including improved job satisfaction, organizational loyalty, work engagement, and career development [22,24].

Emerging adults with positive peer relationships (high trust and communication and low alienation) are more likely to exhibit more frequent prosocial behaviors [25]. Their prosocial skills may be practiced, strengthened, and consolidated into self-concepts [26]. Peer relationships help new employees acquire knowledge, gain support, and reduce stress [5]. Positive peer attachment at work leads to increased frequency and intensity of proactive socialization behaviors [27]. New employees can gain information through communication to reduce tension caused by uncertainty and unpredictability [28]. The work environment with trust also contributes to the career development of new employees [29]. Individuals who perceive alienation or conflict with their peers tend to exhibit negative emotions, which may lead to deviation behaviors [25]. When new employees are rejected or treated indifferently when entering the organization, they may feel frustrated and lost [30]. Exclusion can adversely affect the psychological state and behaviors of new employees, and impair their socialization [31]. Therefore, research hypotheses 1.1, 1.2, and 1.3 are proposed:

**H1.1:**  *Peer trust positively affects proactive socialization behaviors.*

**H1.2:**  *Peer communication positively affects proactive socialization behaviors.*

**H1.3:**  *Peer alienation negatively affects proactive socialization behaviors.*

Core self-evaluation is an individual basic and enduring assessment of values and abilities [32], which has four characteristics: general self-efficacy, self-esteem, emotional stability, and control locus [33]. High core self-evaluation is often accompanied by high self-awareness and strong adaptability, leading to a certain control over the environment; however, individuals with low core self-evaluations usually have lower self-esteem and limited confidence in their abilities, and often feel helpless in unfamiliar environments [34]. Secure peer attachments are more likely to lead to the development of adaptive positive traits, perceived mutual understanding, and emotional support in the area of intimacy, which may alleviate psychological and social adjustment difficulties [35]. Trust, sharing, and interactions in peer groups promote the spread of security, reinforce positive behaviors, and contribute to healthy self-esteem [36]. Individuals with higher core self-evaluations have positive thoughts and traits that affect career development, are more likely to adapt to their new jobs through self-improvement strategies, and can also improve sustainable adaptation and maintain employability [37,38,39]. When peers establish reciprocal communication, newcomers can quickly acquire job task knowledge and role clarity, and show more positive and proactive behaviors in the organization [40]. The quality of peer communication is negatively correlated with social avoidance, which reduces the perceived quality of friendship and increases peer rejection, resulting in poor socialization [41]. Peer alienation is thought to be an insecure attachment relationship that lowers core self-evaluation and leads to perceived job insecurity, which is a significant source of employee stress [42]. Loneliness and emotional exhaustion due to alienation can significantly predict abnormal behaviors in the workplace [43]. Therefore, research hypotheses 2.1, 2.2, and 2.3 are proposed:

**H2.1:**  *Core self-evaluation has a mediating effect between peer trust and proactive socialization behaviors.*

**H2.2:**  *Core self-evaluation has a mediating effect between peer communication and proactive socialization behaviors.*

**H2.3:**  *Core self-evaluation has a mediating effect between peer alienation and proactive socialization behaviors.*

Knowing oneself and knowing others are as indispensable to human beings as the ability to recognize objective things or sounds [44]. Unlike traditional intelligence, social intelligence describes people’s abilities to understand others in interpersonal relationships, to communicate well with others, and behave appropriately, which involve the interaction between personal desires, needs, attitudes, emotional states, and perceiving others [45]. Social intelligence is considered to be the cognitive basis of personality, which is continuously formed in the process of life practice and adaptation to the environment [46]. Numerous research studies have confirmed that social intelligence is more predictive of individual life success and positive perception than other intelligence (such as academic intelligence, emotional intelligence, etc.). Children with high social intelligence report higher scores in peer acceptance, environmental adaptation, and academic development, and also have higher subjective well-being [47,48]. In the workplace, employees with high social intelligence are more prominent in the interview process, adapt more easily to the organization, demonstrate higher leadership skills, and have higher efficiency at work [49,50]. High social intelligence is also significantly correlated with teamwork relationships and innovation within organizations, and can effectively predict managerial performances [51,52]. Social intelligence can help individuals to increase self-awareness while constantly in contact with peers, and further enhance the impact of positive peer relationships on individuals’ self-affirmation [53]. At the same time, because of their high interpersonal sensitivity [54], they will actively seek to improve interpersonal relationships, thereby weakening the impact of negative peer relationships on core self-evaluation. Therefore, research hypotheses 3.1, 3.2, and 3.3 are proposed:

**H3.1:**  *Social intelligence has a positive moderating effect between peer trust and core self-evaluation.*

**H3.2:**  *Social intelligence has a positive moderating effect between peer communication and core self-evaluation.*

**H3.3:**  *Social intelligence has a negative moderating effect between peer alienation and core self-evaluation.*

Social intelligence reflects the personal ability to adopt strategies and methods in order to achieve corresponding goals and obtain positive development in a specific social situation [55]. Individuals with high social intelligence can use their good interpersonal abilities and analysis capabilities to offset the negative effects of some unfavorable environments [56,57]. When new employees first enter an organization, they generally feel high psychological pressure due to their unfamiliarity with the environment [58]. Social intelligence can help individuals proactively make connections within an organization. Positive peer attachment has a significant positive relationship with individual self-identity, while social intelligence is a favorable condition for realizing proactive behaviors. Individuals are more aware of their own motivations, understand the intentions of others, and show more social behaviors [46,59]. The perceived trust of new employees plays an important role in stimulating creativity and reducing the pressure on new employees during the adaptation period [60]. Interpersonal trust increases employees’ sense of responsibility and belonging, thereby making efforts to integrate into the organization [61]. Research studies have also shown that there is a linear positive correlation between communication skills and social intelligence level [62]. Higher social intelligence is accompanied by improved communication skills and positive traits [63]. Even when faced with alienated peer relationships, people with high social intelligence are usually good at coordinating resources, actively responding to challenges in inexperienced environments, and using appropriate strategies to deal with threats so as to integrate into the organization as soon as possible [64]. Therefore, research hypotheses 4.1, 4.2, and 4.3 are proposed:

**H4.1:**  *Social intelligence positively moderates the indirect effects between peer trust, core self-evaluation, and proactive socialization behavior.*

**H4.2:**  *Social intelligence positively moderates the indirect effects between peer communication, core self-evaluation, and proactive socialization behavior.*

**H4.3:**  *Social intelligence negatively moderates the indirect effects between peer alienation, core self-evaluation, and proactive socialization behavior.*

## 3. Method

### 3.1. Procedure

This study investigated the work statuses of college students one year after graduation through a questionnaire survey, and explored the impact of peer attachment on individual proactive socialization behavior. The data are mainly from coastal cities in eastern China. The survey covered peer attachment, core self-evaluation, proactive socialization behavior, social intelligence, and control variables. Data were collected in two time periods. Time 1: Peer attachment, core self-evaluation, and control variables were measured. Time 2: Proactive socialization behavior and social intelligence were measured one month later. Respondents were informed of the study’s purpose and then began participating in the online survey. All questionnaires were anonymous and guaranteed to be used only for academic research. Respondents were allowed to stop answering at any time. All incomplete questionnaires were considered invalid; 800 questionnaires were distributed through convenience sampling and 477 questionnaires were returned with a recovery rate of 59.6%. Finally, 414 questionnaires were valid with an effective recovery rate of 51.8%.

### 3.2. Participants

There were 234 males (56.5%) and 180 females (43.5%) in the valid sample. The gender ratio was relatively balanced; All respondents were between the ages of 20 and 30 with an average age of 23.9. There were 389 respondents (94%) between the ages of 21 and 25, and 25 respondents (6%) over the age of 26 in the valid sample. This is basically consistent with the age distribution of undergraduate graduates in Chinese universities. Moreover, 356 respondents (86%) are currently employed in full-time positions, and 58 respondents (14%) work part-time; 222 graduates (53.6%) majored in liberal arts and 192 graduates (46.4%) majored in science and engineering. Respondents had a balanced distribution of education majors. The current industries of the respondents are very diverse, including manufacturing (24.5%), service (26.4%), finance (11.3%), education (9.7%), public service (6.1%), construction (3.9%), and other industries (18.1%).

### 3.3. Measures

All the scales in this survey were self-reported, which were measured with Likert’s five-point scale (from 1 complete disagreement to 5 complete agreement).

*Peer attachment*

Peer trust emphasizes mutual trust and respect for the needs and desires between peers; peer communication refers to the perceived quality of participation, responsiveness, and state of verbal communication; peer alienation involves feelings of anger, isolation, and separation from peers [12]. Peer attachment was measured based on the study by Armsden and Greenberg [12]. There were 25 items in total. The internal consistency coefficients of peer trust (e.g., *I can trust my friends*), peer communication (e.g., *I will tell my friends about my troubles*), and peer alienation (e.g., *I still feel isolated when I stay with my friends*) were 0.867, 0.891, and 0.837, respectively.

*Core Self-evaluation*

Core self-evaluation is defined as an individual’s basic and enduring self-assessment of worth and abilities [32]. Core self-evaluation (e.g., *I can determine what will happen in my life*) was measured based on the study by Judge et al. [65]. There were 12 items in total and the internal consistency coefficient was 0.902.

*Proactive Socializing Behavior*

Proactive socializing behavior refers to proactive social behaviors that new members may take to gain a more accurate understanding of the organization, e.g., acquiring information on organizational policies and building social networks within companies [5]. Proactive socialization behavior (e.g., *I will try to build a good relationship with my supervisors*) was measured based on the study by Ashford and Black [5]. There were 21 items in total and the internal consistency coefficient was 0.898.

*Social Intelligence*

Social intelligence describes one’s ability to understand others in interpersonal relationships, communicate well with others, and behave appropriately [45]. Social intelligence (e.g., (*I can predict how other people will react to my behaviors*) was measured based on the study by Suseno et al. [66]. There were 21 items in total and the internal consistency coefficient was 0.830.

*Control Variables*

In this study, gender, age, major, and work status (full-time or part-time) were measured as control variables. Amos 26, SPSS24 and Process 4.0 were used to analyze the data.

## 4. Results

### 4.1. Confirmatory Factor Analysis

Firstly, the discriminant validity and the common method bias were verified through confirmatory factor analysis. The model fit in the six-factor model (peer trust, peer communication, peer alienation, core self-evaluation, proactive socialization behavior, and social intelligence) was acceptable ($\chi^{2}/df$ = 1.760, CFI = 0.967, TFI = 0.959, GFI = 0.931, RMR = 0.040, RMSEA = 0.043), which means that the six-factor model in this study had good discriminant validity. However, the model fit of the one-factor model was far from acceptable ($\chi^{2}/df$ = 11.289, CFI = 0.519, TFI = 0.422, GFI = 0.600, RMR = 0.109, RMSEA = 0.158). There was no serious common method deviation in the study.

### 4.2. Correlation Analysis

The mean, standard deviation, correlation coefficient, and square root of AVE are reported in Table 1. After controlling age, gender, status, and major, all relevant variables in the study were significantly correlated. Peer trust (r = 0.158, *p* < 0.01) and peer communication (r = 0.111, *p* < 0.01) were positively related to core self-evaluation. Peer trust (r = 0.269, *p* < 0.01) and peer communication (r = 0.307, *p* < 0.01) were positively related to proactive socialization behavior. Peer alienation was negatively related to core self-evaluation (r = −0.146, *p* < 0.01) and proactive socialization behavior (r = −0.296, *p* < 0.01). Core self-evaluation was positively related to proactive socialization behavior (r = 0.280, *p* < 0.01). Social intelligence was positively related to peer trust (r = 0.410, *p* < 0.01), peer communication (r = 0.397, *p* < 0.01), core self-evaluation (r = 0.308, *p* < 0.01), and proactive socialization behavior (r = 0.516, *p* < 0.01). Social intelligence was negatively related to peer alienation (r = −0.403, *p* < 0.01).

### 4.3. Hypotheses Tests

The impacts of peer attachment on proactive socialization behavior, and the mediating effects of core self-evaluation between peer attachment and proactive socialization behavior were tested through hierarchical regression. In Table 2, Model 1 verifies the impact of peer trust on proactive socialization behavior. When controlling age, gender, status, and major, peer trust (β = 0.267, *p* < 0.001) had a significant positive impact on proactive socialization behavior. Model 4 verifies the influence of peer communication on proactive socialization behavior. When controlling age, gender, status, and major, peer communication (β = 0.311, *p* < 0.001) had a significant positive impact on proactive socialization behavior. Model 7 verifies the influence of peer alienation on proactive socialization behavior. When controlling age, gender, status, and major, peer alienation (β = −0.277, *p* < 0.001) had a significant negative impact on proactive socialization behavior. Model 10 verifies the influence of core self-evaluation on proactive socialization behavior. When controlling age, gender, status, and major, core self-evaluation (β = 0.272, *p* < 0.001) had a significant positive influence on proactive socialization behavior. Individuals with higher peer trust and peer communication were more likely to exhibit proactive socialization behaviors. Peer alienation reduced individual proactive socialization behaviors. Therefore, research hypotheses 1.1, 1.2, and 1.3 have been verified.

Models 1 to 3 verify the mediating role of core self-evaluation between peer trust and proactive socialization behavior. Peer trust significantly affected proactive socialization behavior (β = 0.267, *p* < 0.001) and core self-evaluation (β = 0.181, *p* < 0.001). However, due to the intervention of core self-evaluation(β = 0.236, *p* < 0.001), the influence of peer trust on proactive socialization behavior decreased (β = 0.224, *p* < 0.001). Core self-evaluation had a partial mediating effect between peer trust and proactive socialization behaviors. Models 4 to 6 verify the mediating role of core self-evaluation between peer communication and proactive socialization behavior. Peer communication significantly affected proactive socialization behavior (β = 0.311, *p* < 0.001) and core self-evaluation (β = 0.125, *p* < 0.001). However, due to the intervention of core self-evaluation (β = 0.240, *p* < 0.001), the influence of peer communication on proactive socialization behavior decreased (β = 0.281, *p* < 0.001). Core self-evaluation had a partial mediating effect between peer communication and proactive socialization behaviors. Models 7 to 9 verify the mediating role of core self-evaluation between peer alienation and proactive socialization behavior. Peer alienation significantly affected proactive socialization behavior (β = −0.277, *p* < 0.001) and core self-evaluation (β = −0.145, *p* < 0.001). However, due to the intervention of core self-evaluation (β = 0.237, *p* < 0.001), the influence of peer alienation on proactive socialization behavior decreased (β = −0.243, *p* < 0.001). Core self-evaluation had a partial mediating effect between peer alienation and proactive socialization behaviors. Therefore, research hypotheses 2.1, 2.2, and 2.3 have been verified.

The moderating role of social intelligence between peer attachment and core self-evaluation and the moderated mediating role of social intelligence between peer attachment, core self-evaluation, and proactive socialization behavior were examined through Process 4.0. The results are shown in Table 3.

Firstly, the moderating role of social intelligence between peer trust and core self-evaluation is discussed. The 95% confidence interval(CI) of the moderating effect (b = 0.626, SE = 0.087) was [0.455, 0.797]. Social intelligence had a moderating effect between peer trust and core self-evaluation since the interval did not pass zero. At a low level of social intelligence (M − 1SD), the 95% CI of the moderating effect between trust and core self-evaluation (b = −0.176, SE = 0.064) was [−0.309, −0.044], and the interval did not pass zero. Therefore, the moderating effect of social intelligence does exist at a low level of peer trust. At a high level of social intelligence (M + 1 SD), the 95% CI of the moderating effect between peer trust and core self-evaluation (b = 0.418, SE = 0.079) was [0.263, 0.574], and the interval did not pass zero. High social intelligence can enhance the influence of peer trust on core self-evaluation. When social intelligence was considered in the indirect influence of peer trust on proactive socialization behavior through core self-evaluation (b = 0.126, SE = 0.045), the 95% CI was [0.049, 0.221], the interval did not pass zero, so social intelligence had a moderated mediating effect on the relationship between peer trust, core self-evaluation, and proactive socialization behavior. Social intelligence may enhance the indirect influence of peer trust on proactive socialization behavior through core self-evaluation. Therefore, research hypotheses 3.1 and 4.1 have been verified.

Secondly, the moderating effect of social intelligence between peer communication and core self-evaluation is discussed. The 95% CI of the moderating effect (b = 0.489, SE = 0.088) was [0.316, 0.662]. Social intelligence had a moderating effect between peer communication and core self-evaluation since the interval did not pass zero. At a low level of social intelligence (M − 1 SD), the 95% CI of the moderating effect between peer communication and core self-evaluation (b = −0.189, SE = 0.066) was [−0.319, −0.059], and the interval did not pass zero. Therefore, the moderating effect of social intelligence exists at a low level of peer communication. At a high-level of social intelligence (M + 1 SD), the 95% CI of the moderating effect between peer communication and core self-evaluation (b = 0.275, SE = 0.078) was [0.121, 0.429], and the interval did not pass zero. High social intelligence can enhance the influence of peer communication on core self-evaluation. When social intelligence was taken into account in the indirect influence of peer communication on proactive socialization behavior through core self-evaluation (b = 0.100, SE = 0.041), the 95% CI was [0.032, 0.192], the interval did not pass zero, so social intelligence had a moderated mediating effect on the relationship between peer communication, core self-evaluation, and proactive socialization behavior. Social intelligence may enhance the indirect influence of peer communication on proactive socialization behavior through core self-evaluation. Therefore, research hypotheses 3.2 and 4.2 have been verified.

Finally, the moderating effect of social intelligence between peer alienation and core self-evaluation is discussed. The 95% CI of the moderating effect (b = −0.294, SE = 0.080) was [−0.452, −0.136]. Social intelligence had a moderating effect between peer alienation and core self-evaluation since the interval did not pass zero. At a low level of social intelligence (M − 1 SD), the 95% CI of the moderating effect between peer alienation and core self-evaluation (b = 0.110, SE = 0.064) was [−0.017, 0.236], and the interval did pass zero. Therefore, the moderating effect of social intelligence did not exist at a low level of peer alienation. At a high level of social intelligence (M + 1 SD), the 95% CI of the moderating effect between peer alienation and core self-evaluation (b = −0.170, SE = 0.065) was [−0.298, −0.042], and the interval did not pass zero. A high level of social intelligence can weaken the influence of peer alienation on core self-evaluation. When social intelligence was taken into account in the indirect influence of peer alienation on proactive socialization behavior through core self-evaluation (b = −0.059, SE = 0.031), the 95% CI was [−0.129,−0.009], and the interval dose did not pass zero, so social intelligence had a negative moderated–mediating effect on the relationship between peer alienation, core self-evaluation, and proactive socialization behavior. Social intelligence may weaken the indirect influence of peer alienation on proactive socialization behavior through core self-evaluation. Therefore, research hypotheses 3.3 and 4.3 have been verified.

## 5. Discussions and Conclusions

There is a huge difference between campus life and the workplace. Some college graduates are unable to adapt to their new roles, resulting in a high frequency of resignations after graduation. In the process of changing social roles, it is very important for emerging adults to actively understand social realities and integrate into organizations [16]. The research uses a questionnaire to explore the situation of college graduates after one year of work. Statistical analysis results show that peer attachment significantly affects individual proactive socialization behaviors. Core self-evaluation has a mediating effect between peer attachment and individual proactive socialization behaviors. Peer trust and peer communication can improve individual proactive socialization behaviors by enhancing core self-evaluation. However, peer alienation may reduce core self-evaluation and inhibit individual proactive socialization behavior in the workplace. Social intelligence plays a moderating role between peer attachment and core self-evaluation. High social intelligence may enhance the influence of peer communication on core self-evaluation and weaken the influence of peer alienation on core self-evaluation. Social intelligence also has a moderated mediating role between peer attachment, core self-evaluation, and proactive socialization behavior. High social intelligence may enhance the indirect influence of peer trust and peer communication on proactive socialization behavior through core self-evaluation and weaken the indirect influence of peer alienation on proactive socialization behavior through core self-evaluation.

For young employees, peer influence is increasingly important [8]. Numerous previous studies have explored the value of peer relationships in the workplace, and support from colleagues and supervisors can help employees grow rapidly [67]. This study focuses on the personal social statuses of young employees. Gaining the trust and recognition of peers, both in an individual’s social network and within the organization, leads to improved positive perceptions of one’s self and enhanced self-identity, which stimulates proactive behaviors aimed at adapting to the organization. On the contrary, estranged peer relationships will reduce an individual’s self-confidence and weaken his/her motivation to take proactive socialization behaviors [41]. In this process, social intelligence is an important boundary condition and a good predictor of personnel selection, which can help organizations identify highly adaptable and resilient employees [49]. High social intelligence is more likely to lead to the formation of positive peer relationships due to better interpersonal communications and change the temporary alienation state, thereby preventing negative self-perceptions and reducing proactive behaviors [63,64].

Organizational socialization can help employees better integrate into an organization and start work quickly [24]. Besides the organization’s supportive policies and measures, the proactive socialization behaviors of new employees are more expected and valued [4]. Firstly, organizations should pay attention to candidates’ peer attachments and social intelligence levels and identify applicants with these positive behavioral tendencies in the recruiting process. Through interviews and selection tests, recruiters can learn about a candidate’s peer relationships and predict his/her future performance within the organization. When individuals demonstrate trust and communication with their peers, rather than alienation, they tend to have higher self-awareness and may take a proactive approach to integrate into the organization. Social intelligence as a positive personal trait should also be considered as one of the selection criteria. While actively adapting to the organizational environment, people with high social intelligence can also help to create a harmonious working atmosphere within the organization.

Secondly, managers should strive to create a positive team atmosphere and corporate culture, and build formal and informal platforms to promote employee cooperation. The attitudes and behaviors of superiors have a greater impact on the formation of psychological contracts for new employees, which will affect their future on-the-job behaviors in the organization [68]. Organizations can encourage senior employees to support new employees, strengthen interaction communications, and enhance mutual trust in the cooperation. At the same time, managers can also try to stimulate informal communication between team members, specifically through team-building activities; the unfamiliar relationship between employees can be changed and communication topics accumulated. Organizations can also enrich experience-sharing and empathy among colleagues by hosting various themed dinners and outreach events. Enhanced positive attachments among employees can prevent alienated relationships caused by a lack of communication, thereby promoting proactive socialization behaviors for new employees.

Finally, managers should emphasize the positive guidance of employees’ core self-evaluations, such as management development training, job rotation, reward sharing, etc. Learning can improve employees’ communication skills and abilities to deal with complex tasks. Challenging and diverse work assignments can help employees integrate into the organization soon and increase their self-confidence. Sharing can enhance mutual communication and trust, which will indirectly improve the positive orientation of individual core self-evaluation. Of course, there will inevitably be disputes and disagreements in the interactions between organization members, especially for newly hired employees, due to their unfamiliarity with the environment and work tasks, there will be high work pressure and difficulties in cooperating with colleagues [69]. In this process, managers should play guiding and coordinating roles, encourage employees to learn from each other, and increase understanding and tolerance.

In addition to relying on the organizational environment and the correct guidance of managers, new employees should also actively strive to promote their own socialization processes from personal perspectives. High-quality interpersonal relationships are essential for personal growth [20]. New employees should make full use of their personal social resources and strengthen communications with classmates and friends. They can share the problems encountered in the workplace with their peers, which will not only generate emotional support but also solve existing difficulties through exchanging information with each other and accelerating the process of integration into the organization. At the same time, individuals should actively participate in formal and informal communications with new colleagues in the workplace, and familiarize themselves with the new environment and rules at work. Positive interactions among colleagues are conducive to the establishment of harmonious working relationships, enhance confidence in cooperation, and can improve an individual’s social intelligence and core self-evaluation. Integrating into the organization and being accepted by the organization’s members will further increase the individual’s willingness to exhibit proactive socialization behaviors.

The study has certain limitations. Chinese culture advocates collectivism and people value their relationships with each other [66]. Relationships with colleagues and friends have serious impacts on an individual’s self-perception and behaviors, thus leading to the formation of a higher level of peer attachment. This may be different in countries characterized by individualism, so the conclusions of this study may have certain external validity problems. IPPA is a scale developed for adolescent peer attachment. Relatively few studies have used it to measure peer attachment among emerging adults [26,70]. Although the data showed acceptable reliability/validity in this study, which is not significantly different from the adolescent sample. More targeted measurement scales will be considered in future research. In the questionnaire survey, we limited the coverage of demographic variables, such as age distribution, industry distribution, major, etc., but not personal marital statuses, family structures, seniority, etc. These factors may also have certain impacts on the results of the study and can be considered as control variables in future studies.

Peer attachment can affect proactive socialization behaviors through core self-evaluation. Social intelligence has a moderating effect on the relationships among peer attachments, self-core evaluation, and proactive socialization behaviors. The formation mechanism of proactive socialization behavior is very complex, and more personal, organizational, and situational factors are worthy of further exploration. In future research, we will consider using the experimental method to further verify the influence of peer relationships on proactive socialization behaviors, and explore the synergistic effects of organizational factors (organizational culture, organizational structure, team type, etc.) and personal factors (personal traits, interpersonal relationships, leadership behaviors, etc.). We hope that we can continue to discuss the situational conditions for the formation of a win–win situation between new employees and organizations.

## Figures and Tables

**Table 1:** Correlation statistics (N = 414).

	Mean	SD	1	2	3	4	5	6	7	8	9	10
Gender	1.43	0.496	-									
Age	1.56	0.660	0.289 **	-								
Status	1.14	0.348	0.137 *	0.575 **	-							
Major	1.57	0.614	0.328 **	0.543 **	0.105 *	-						
PT	3.67	0.602	0.238 *	0.158 **	0.006	0.296 **	(0.762)					
PC	3.58	0.630	0.248 **	0.170 **	0.063	0.248 **	0.798 **	(0.780)				
PA	2.49	0.718	0.046	−0.092	−0.004	−0.085	−0.408 **	−0.442 **	(0.719)			
CS	2.96	0.744	−0.039	0.020	0.044	0.024	0.158 **	0.111 **	−0.146 **	(0.734)		
PB	3.44	0.614	−0.031	0.123 *	0.042	0.158 **	0.269 **	0.307 **	−0.296 **	0.280 **	(0.731)	
SI	3.23	0.475	−0.032	0.152 **	0.017	−0.187 **	0.410 **	0.397 **	−0.403 **	0.308 **	0.516 **	(0.881)

Note: ** *p* < 0.01, * *p* < 0.05; PT: peer trust; PC: peer communication; PA: peer alienation; CS: core self-evaluation; PB: proactive socialization behavior; SI: social intelligence.

**Table 2:** Results of the hierarchical regression analysis (N = 414).

	Model 1	Model 2	Model 3	Model 4	Model 5	Model 6	Model 7	Model 8	Model 9	Model 10
	PB	CS	PB	PB	CS	PB	PB	CS	PB	PB
Gender	−0.145	−0.086	−0.125 *	−0.159 *	−0.079	−0.140 *	−0.076	−0.043	−0.066	−0.086
Age	0.068	−0.026	0.074	0.064	−0.027	0.071	0.033	−0.044	0.044	0.075
Status	0.012	0.069	−0.004	−0.002	0.060	−0.017	0.018	0.070	0.001	−0.016
Major	0.088	0.005	0.087	0.098	0.027	0.091	0.139	0.042	0.129	0.140
PT	0.267 ***	0.181 ***	0.224 ***							
PC				0.311 ***	0.125 ***	0.281 ***				
PA							−0.277 ***	−0.145 ***	−0.243 ***	
CS			0.236 ***			0.240 ***			0.237 ***	0.272 ***
ΔR${}^{2}$	0.099	0.035	0.153	0.123	0.020	0.180	0.111	0.026	0.166	0.109
F	8.963 ***	2.918 ***	120.227 ***	11.520 ***	1.640 ***	14.991 ***	10.205 ***	2.185 ***	13.509 ***	10.028 ***

Note: *** *p* < 0.001, * *p* < 0.05; PT: peer trust; PC: peer communication; PA: peer alienation; CS: core self-evaluation; PB: proactive socialization behavior.

**Table 3:** Moderation Effect (N = 414).

Moderating Effect					Moderated Mediating Effect					
Variables	SI	Effect	SE	*p*	95% CI		Effect	SE	95% CI	
					Lower	Upper			Lower	Upper
PT	Int	0.626	0.087	0.000	0.455	0.797	0.126	0.045	0.049	0.221
	L(M− 1 SD)	−0.176	0.064	0.009	−0.309	−0.044				
	H(M + 1 SD)	0.418	0.079	0.000	0.263	0.574				
PC	Int	0.489	0.088	0.000	0.316	0.662	0.100	0.041	0.032	0.192
	L(M − 1 SD)	−0.189	0.066	0.004	−0.319	−0.059				
	H(M + 1 SD)	0.275	0.078	0.000	0.121	0.429				
PA	Int	−0.294	0.080	0.000	−0.452	−0.136	−0.059	0.031	−0.129	−0.009
	L(M − 1 SD)	0.110	0.064	0.089	−0.017	0.236				
	H(M + 1 SD)	−0.170	0.065	0.010	−0.298	−0.042				

Note: PT: peer trust; PC: peer communication; PA: peer alienation; CS: core self-evaluation; PB: proactive socialization behavior; SI: social intelligence; L: lower level; H: higher level; CI: confidence interval.

## Data Availability

The data presented in this study are available upon request from the corresponding author.

## Abbreviations

The following abbreviations are used in this manuscript:

PT	peer trust
PC	peer communication
PA	peer alienation
CS	core self-evaluation
PB	proactive socialization behavior
SI	social intelligence
